# Single melatonin injection enhances the testicular artery hemodynamic, reproductive hormones, and semen parameters in German shepherd dogs

**DOI:** 10.1186/s12917-022-03487-y

**Published:** 2022-11-14

**Authors:** Ali Salama, Elshymaa A. Abdelnaby, Ibrahim A. Emam, Mohamed Fathi

**Affiliations:** 1 Theriogenology Department, Faculty of Veterinary Medicine, Cairo University, Giza, Egypt; 2 Department of Surgery, Anesthesiology and Radiology, Faculty of Veterinary Medicine, Cairo University, Giza, Egypt

**Keywords:** Canine, Doppler, Melatonin, Sperm, Testicular artery, Volume

## Abstract

This study aimed to determine the effects of melatonin administration on testicular vascular perfusion in relation to steroid hormones and semen characteristics in dogs. The study included 12 normospermic German shepherd dogs (weighed 35 ± 0.5 kg and aged 4 ± 0.5 years). Males received a single melatonin administration (melatonin dimethyl sulfoxide + corn oil via subcutaneous route; MEL; *n* = 6), while the rest of the animals served as controls (dimethyl sulfoxide + corn oil; Control; *n* = 6). Males were subjected to routine examination on days -15, 0, 15, 30, 45, and 60. All examined dogs were subjected to Doppler screening, semen collection, and blood sampling. The MEL group showed a significant (*P* < 0.05) elevation in semen volume, concentration, percentage of sperm motility, and total sperm × 10^6^ / ejaculate compared to other control males. Doppler indices as resistance (RI) and pulsatility (PI) indices declined (*P* < 0.05) from D 30 (1.02 ± 0.01) until day 60 (0.87 ± 0.02) of treatment. In MEL males, the peak systolic point of velocity (PSV; cm/sec) of the testicular artery elevated (*P* < 0.05) on day 60 (20.15 ± 0.99) compared to its value on day 0 (17.39 ± 1.84). On D 60, the levels of testosterone (T), estradiol 17-ß (E2), and nitric oxide (NO) elevated (*P* < 0.05). A negative correlation was detected between testicular volume, scrotal circumference (SC), T levels, Doppler indices, and velocities. In conclusion, single melatonin administration could improve testicular vascularization via increasing Doppler velocities and intratesticular colored areas. In addition, it could improve semen picture and steroids (T and E2) and nitric oxide.

**Supplementary Information:**

The online version contains supplementary material available at 10.1186/s12917-022-03487-y.

## Introduction

Canines can be bred so long as they are predominantly fertile, and maintaining this species's reproductive ability remains an issue of interest [1]. A male's potential fertility can be predicted by evaluating the quality of the semen before the beginning of its reproductive life [2]. Palpation is regarded as an ineffective approach for measuring the testicular parenchyma [3]. B- mode ultrasonographic examination of the male reproductive organs has gained popularity since it is a simple method for evaluating biometric parameters but lacks information about the organ vasculature [3]. The testicular vasculature system is the primary conduit for nutrients and other hormones to and from the testis [4]. Numerous animal species have employed testicular blood flow measurements to assess the functionality of the testicles [5, 6]. Color Doppler ultrasonographic technology has been demonstrated for the assessment of male fertility in humans via the determination of testicular functionality [7, 8] and has been applied in veterinary medicine to a variety of species, including dogs [9–11], rams [12, 13], and stallions [14, 15]. This ultrasound tool may be a good predictor of semen quality in humans [8] and dogs [16]. Testing hemodynamic alterations using pulsed Doppler ultrasound is a crucial step in diagnosing various cases of testicular dysfunction because testicular blood flow is essential for steroidogenic and spermatogenic processes in farm animals [14]. Since basal nitric oxide controls testicular hemodynamics [17], nitric oxide (NO) is regarded as a free radical associated with the erection process [18]. Additionally, testosterone evaluation is essential for identifying subfertility affections, which are problems with fertility [19].

The pineal gland secretes melatonin. Melatonin is a tryptophan derivative that is widely recognized as a potent antioxidant [20, 21]. Melatonin plays a vital role in stimulating antioxidant enzymes in the reproductive system [22, 23], as well as in all body systems, against free radicals [24]. In men, melatonin regulates GnRH and LH secretion, testosterone production, and testicular maturation, thereby preventing environmental toxin-induced testicular damage [25, 26]. Several previous animal studies have demonstrated melatonin's positive role in reproductive performance [22, 27, 28]. To date, the effect of subcutaneous melatonin injection on testicular perfusion is poorly understood in canines. Hence, we hypothesized that examining the effects of melatonin administration could contribute to enhancing semen quality and vascular perfusion. This study determined the impact of melatonin injection on the testicular hemodynamic pattern in relation to steroid hormones and semen quality in dogs.

## Materials and methods

The Faculty of Veterinary Medicine at Cairo University accepted all procedures in this study with an approval number Vet CU 24112020262. This work was performed at the Surgery, Anesthesiology, and Radiology Department, Faculty of Veterinary Medicine-Cairo University (30.0154° N, 31.2120° with a temperature 25–33°C, and relative humidity 55%) between September 1^st^ and October 30^th^, 2020.

### Animals and management

Prior to melatonin administration, all males underwent monthly semen collection and evaluation. Twelve dogs (German Shepherd; normospermic; 35±0.5 kg BW, age: 4±0.5 years) were included in this study, as all males had excellent fertility confirmed by semen collection and semen picture assessment. During the study, all animals were housed indoors with daily exercise and fed commercial food composed of cereals such as rice bran, fats, vegetables, and vitamins, with free access to water all day. All males underwent the clinical examination, followed by ultrasonographic scanning of the male genital organs [29, 30].

### Melatonin injection

Dogs were categorized as males who received a single dose of melatonin: melatonin+ DMSO+ corn oil (MEL group; *n*=6), and dogs that served as the control group (Control group; *n*=6) received only DMSO + corn oil. Melatonin (from Sigma Chemicals) was dissolved in DMSO with corn oil of 1 ml [31] at a final concentration of 18 mg of melatonin/animal by the subcutaneous route with 2 ml of the final mixture (MEL group; *n*=6) as previously done in cats [32]. The drug solubility was approximately 30 mg/ml. Due to its short half-life, melatonin was dissolved in the early morning and administered immediately at day zero [33]. The dose was estimated by mixing 108 mg of melatonin powder in 6 ml of DMSO and extended with an equal volume of corn oil (6 ml).

### Experimental design

**Fig. 1:**
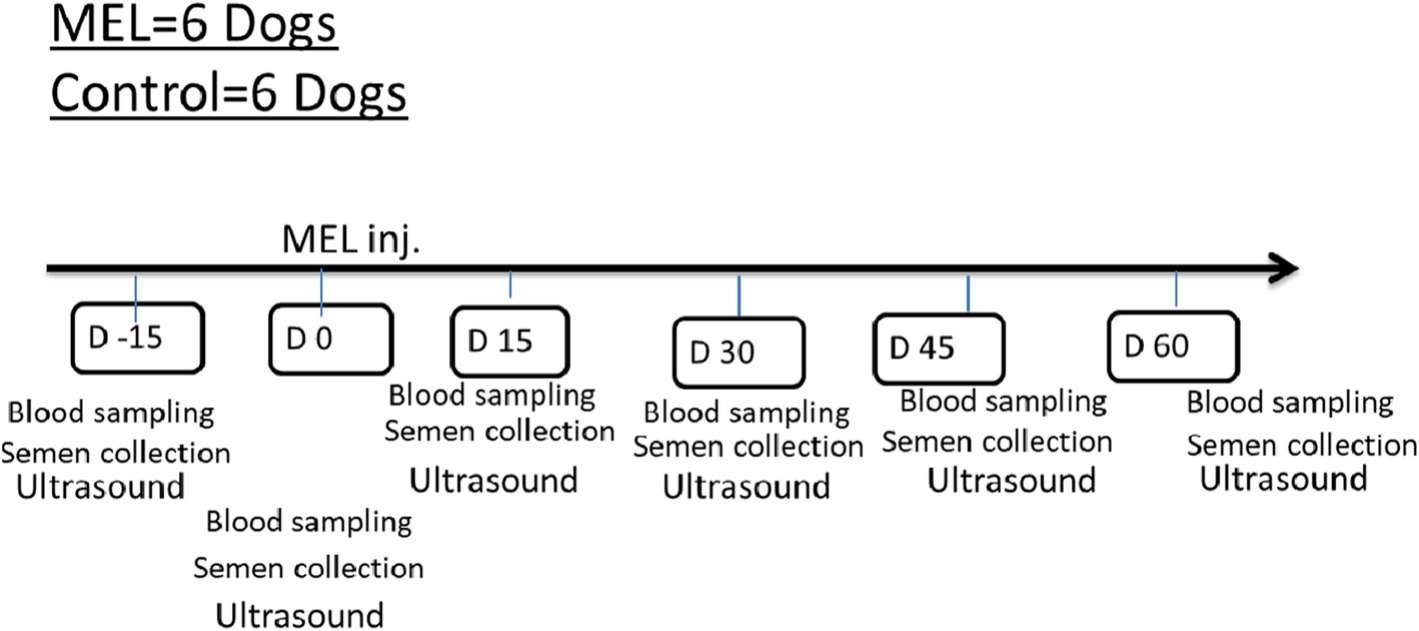
Schedule of periodic assessments, including the day of melatonin injection and the days before and after treatment

Males were subjected to routine examination (every 15 days: 2 times/month) at days -15, 0, 15, 30, 45, and 60 to determine the effect of a single injection of melatonin on testicular hemodynamics and semen pictures compared to other non-injectable animals serving as controls. Dogs were subjected to blood collection, semen collection, and Doppler examination. The assessment was performed on day −15 before melatonin administration to minimize extragonadal contribution (Fig. 1), and the examination was extended to day 60.

### Semen collection and evaluation

This procedure was best carried out in the presence of an estrous bitch. The dog's penis was initially vigorously rubbed through the prepuce at the level of the bulbus glandis until a partial erection appeared. Then, a complete erection was achieved by squeezing the penis between the index inger and thumb, followed by pelvic thrusting. The first two fractions were collected to determine the semen volume (mL), and the obtained semen samples were then sent to the lab for additional examination. All semen samples were collected in falcon tubes (50 ml, one collection every 15 days). In a calibrated tube, direct measurements of the canine semen volume were determined. With the aid of a light microscope and a hemocytometer, the sperm cell concentration was calculated [34]. The sperm cell concentration was used to calculate the total number of sperm in the ejaculate [35]. Immediately after collection, the percentage of sperm motility was subjectively estimated to the nearest 5%, using a phase-contrast microscope with a heated stage (37°C) at 200× magnification [36]. Two observers each examined a sample, and the variation within a sample never exceeded 5%.

### Blood sampling and hormonal assaying

Blood samples were drawn from the male dog's jugular vein and centrifuged at 3000 xg for 15 minutes prior to grey and Doppler assessments. Prior to hormonal analysis, plasma and serum samples were kept at 20°C. Serum samples were utilized to assess nitric oxide (NO), which was previously measured in accordance with the instructions of the commercial kit as serum samples were mixed with an equal amount of Griess reagent and incubated for 10 minutes at room temperature. Plasma samples were used to analyze both testosterone (T) and estradiol 17-ß (E2).

### Scrotal circumference measurement

As previously performed in bucks, the circumference of the entire scrotum was measured by tape in both control and MEL-treated dogs [36].

### Ultrasonographic evaluation

The same operator performed all ultrasound measurements using a lineararray probe. The settings of the Doppler device (EXAGO, France) were optimized as follows: frequency ranged from 7.5 to 10 MHz[37], the pulse repetition frequency (PRF) was 4000 kHz, the Doppler angle of insonation in the spectral mode was 45°±5, the wall filter was 150, and the gate window size was 1 mm [38, 39].

### Measurement of the testicular artery Doppler parameters

The three best continuous waves with complete systolic and diastolic endpoints were measured to determine all Doppler velocimetry measurements, including peak systolic velocity (PSV; cm/sec), end-diastolic velocity (EDV; cm/sec), and both Doppler indices expressed by the resistance index (RI) and pulsatility index (PI) that most commonly used [40, 41]. The distal branch of the testicular artery was identified using image-specific waves [42].

## Evaluation of pampiniform colored area% and testicular volume

The testicular volume could be calculated using the ellipsoid formula: length (L), width (W), and height (H) x0.5236; meanwhile, the pampiniform colored area/pixels divided by the area of the region/pixels was used to calculate the percentage of colored areas in the pampiniform plexus. All frozen images were stored in flash memory for pixel analysis using the Photoshop tool in version CCX64 [43].

### Statistical analysis

Data were assessed using SPSS software (SPSS, 2007). All data are presented as the mean ± standard error of the mean and were first checked for normality by the Shapiro‒Wilk test. The paired t-test was used to compare between two means of the same animal; the right and left sides of the animals. To compare time points within the group and investigate the effects of treatment and time, repeated measures ANOVA was used to examine differences in concentrations, sperm motility %, volume, the total count of sperm per ejaculation, and Doppler parameters. The significant means (*P*<0.05) were separated using Duncan multiple range tests. In MEL dogs, correlation coefficients were calculated between testicular blood flow waveform measurements and semen analytical parameters.

## Results

### Testicular volume assessment

**Fig. 2:**
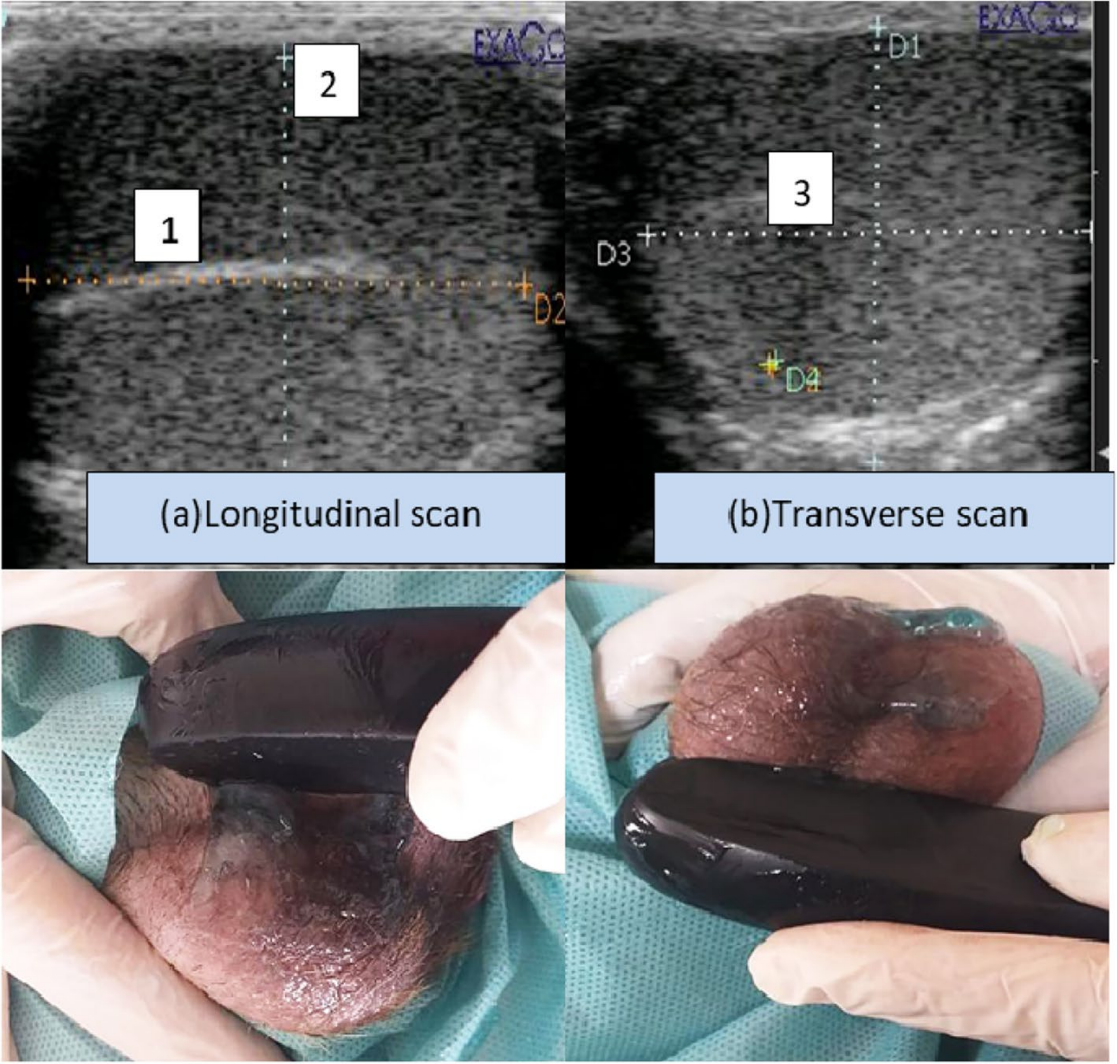
Ultrasonography revealed the testicular dimensions to estimate the testicular volume using the ellipsoid shape formula. Length (1) and height (2) are measured in the longitudinal scan (**a**), while the testicular width (3) is measured in the transverse scan (**b**)

The investigation was completed to examine the impact of melatonin administration throughout various time intervals (days 0, 15, 30, 45, and 60). In dogs, there were no variations between the right and left testicles. Testicular length, width, and height were calculated via grey B- mode to determine the testicular volume, as shown in (Fig. 2a & b).

### Semen characteristics evaluation

From day 30 (7.03 mL± 0.02) until the end of the examination (D 60, 7.65 mL± 0.02), there was an increase (*P* <0.05) in semen volume in the MEL group compared to controls. The MEL dogs showed a substantial increase in sperm concentration from D 15 following melatonin injection (266×10^6^± 40.31/mL) when compared to D 60 (310×10^6^± 15.62/mL). In advance, the total sperm number/ejaculation was increased (*P* <0.05) in the MEL dogs at day 30 after melatonin injection (952×10^6^ spermatozoa/total ejaculated) when compared to day 60 (995×10^6^ spermatozoa/total ejaculated), as depicted in Table 1. At day 30, the sperm motility % (*P*< 0.05) increased from 85.01 ± 3.71 to 90.21 ± 3.55.

**Table 1:** Semen volume, concentration, total sperm × 10^6^/ejaculation, and motility % in dogs treated with melatonin and compared to the control. Data are obtained as mean ± SEM with sample size = 12 × 6 = 72

Days	Semen volume (mL)		Concentration (*10^6^/mL)		Total sperm*10^6^/ejaculation		Motility%	
	**MEL**	**Control**	**MEL**	**Control**	**MEL**	**Control**	**MEL**	**Control**
D-15	4.23 ± 0.01	4.45 ± 0.01	177 ± 32.14	180 ± 42.01	750 ± 12.31	790 ± 3.62	68.21 ± 9.65	70.12 ± 4.21
D 0	4.51 ± 0.02	4.55 ± 0.03	190 ± 15.66	180 ± 47.32	760 ± 19.33	800 ± 45.32	70.11 ± 5.02	70.32 ± 3.21
D 15	5.5 ± 0.11	5.21 ± 0.02	266 ± 40.31^b^	200 ± 18.62^a^	800 ± 15.68	820 ± 45.25	71.19 ± 4.25	75.25 ± 4.55
D 30	7.03 ± 0.02^b^	5.11 ± 0.02^a^	310 ± 25.31^b^	210 ± 28.66^a^	952 ± 40.32^b^	800 ± 18.66^a^	85.51 ± 3.71^b^	75.85 ± 4.08^a^
D 45	7.13 ± 0.01^b^	6.10 ± 0.04^a^	290 ± 15.32^b^	220 ± 41.32^a^	987 ± 25.31^b^	800 ± 28.66^a^	85.56 ± 7.01^b^	80.41 ± 4.82^a^
D 60	7.65 ± 0.02^b^	5.59 ± 0.01^a^	310 ± 15.62^b^	230 ± 47.11^a^	995 ± 15.64^b^	810 ± 47.25^a^	90.21 ± 3.55^b^	75.22 ± 2.65^a^

Means with different (a, b) superscripts are significantly different *P* < 0.05

### Testicular hemodynamics

**Fig. 3:**
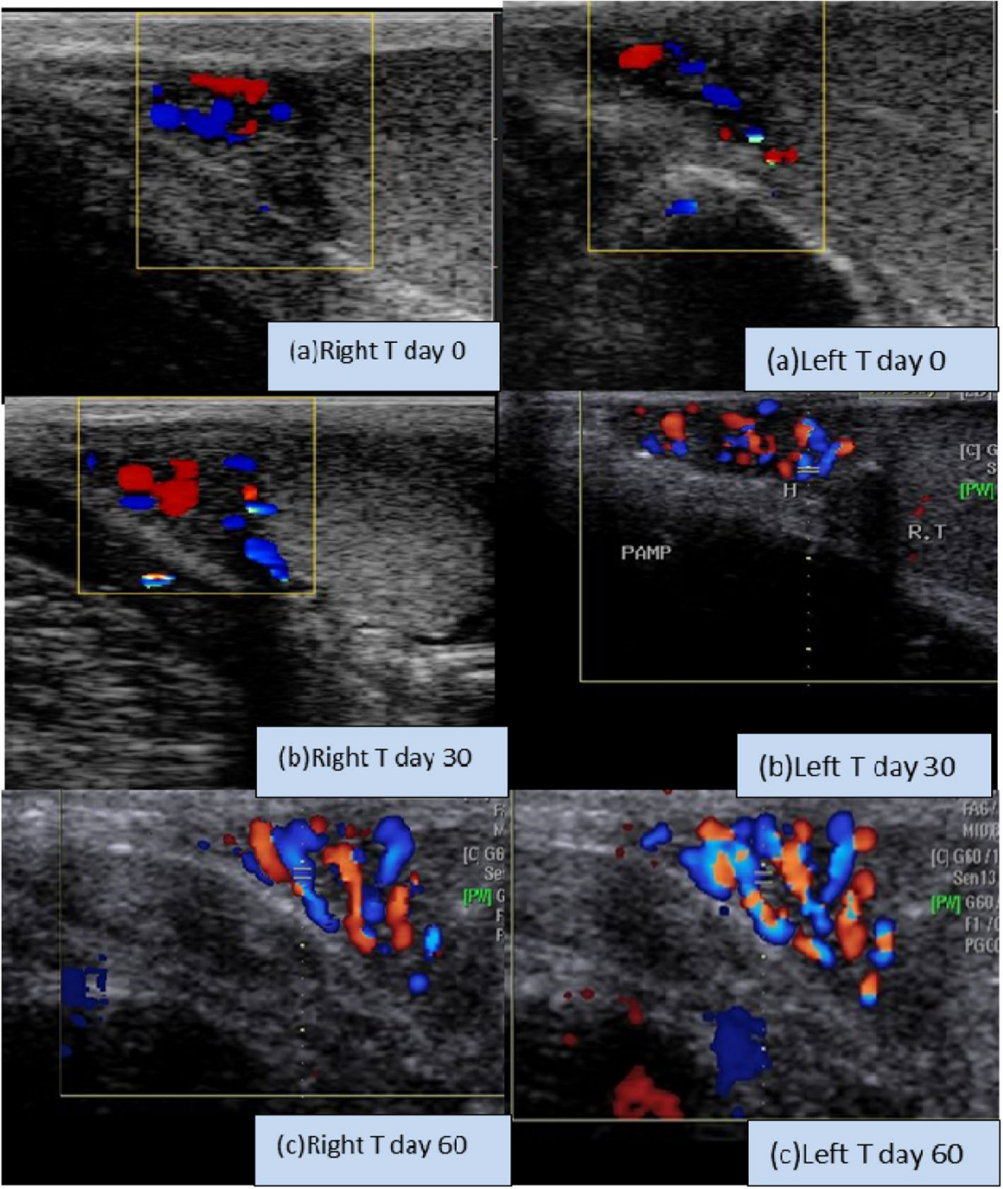
Color and spectral Doppler ultrasonograms showing the elevation in the pampiniform plexus colored area/pixels in both canine testicles at D 0 (**a**), D 30 (**b**) and D 60 (**c**) in the MEL group

Melatonin reduced the main Doppler pulsatility (PI) and resistance (RI) indices from D 30 (1.02±0.0.01 for PI, and 0.51±0.01 for RI) when compared to D 60 (0.87±0.02 for PI and 0.44±0.01 for RI; Table 2). The percentage of the pampiniform colored region (Fig. 3) was elevated (*P*<0.05) from D 30 (81.62±2.91) when compared to D 60 (88.97±7.21) in the MEL group compared to the control group at days 30 and 60 (71.88±3.58 and 76.15±3.81).

**Table 2:** Values of the distal branch of supra testicular artery Doppler indices including pulsatility index(PI),resistance index (RI),testicular volume (cm^3^),and percentage of colored area in the pampiniform plexus in the melatonin treated male compared to the normal dogs. Data are obtained as mean ± SEM with sample size = 12 × 6 = 72

Days	Supra-testicular A PI		Supra-testicular A RI		Testicular volume (cm^3^)		Plexus colored area%	
	**MEL**	**Control**	**MEL**	**Control**	**MEL**	**Control**	**MEL**	**Control**
D-15	1.22 ± 0.01	1.21 ± 0.01	0.88 ± 0.01	0.88 ± 0.02	10.65 ± 0.21	11.03 ± 2.01	70.21 ± 2.55	70.33 ± 6.21
D 0	1.22 ± 0.01	1.22 ± 0.01	0.91 ± 0.01	0.89 ± 0.02	10.69 ± 0.51	11.21 ± 1.36	70.32 ± 3.25	70.28 ± 4.21
D 15	1.16 ± 0.01	1.16 ± 0.01	0.82 ± 0.01	0.88 ± 0.01	11.62 ± 0.09	10.95 ± 0.85	75.21 ± 4.08	73.69 ± 4.88
D 30	1.02 ± 0.01^b^	1.13 ± 0.02^a^	0.51 ± 0.01^b^	0.89 ± 0.01^a^	11.52 ± 0.07	10.99 ± 0.41	81.62 ± 2.91^b^	71.88 ± 3.58^a^
D 45	0.99 ± 0.02^b^	1.16 ± 0.02^a^	0.49 ± 0.01^b^	0.86 ± 0.02^a^	11.62 ± 0.14	11.21 ± 0.33	88.69 ± 5.88^b^	75.36 ± 7.21^a^
D 60	0.87 ± 0.02^b^	1.12 ± 0.01^a^	0.44 ± 0.01^b^	0.85 ± 0.01^a^	11.52 ± 0.88	11.56 ± 0.02	88.97 ± 7.21^b^	76.15 ± 3.81^a^

Means with different (a, b) superscripts are significantly different *P* < 0.05

*A* artery, *PI* pulsatility index, *RI* resistance index, *MEL* melatonin group

## Scrotal circumference, morphometry, Doppler velocities, and hormonal levels on day 0 and day 60

However, melatonin injection did not significantly alter the testicular volume (*P*> 0.05). The distal supra-testicular artery PSV (cm/sec) was elevated significantly (*P*<0.05) at day 60 when compared to day 0 in the MEL males, but its value did not show a marked difference in normal dogs in two-time points (18.09±2.14 on D 0, and 18.32±2.05 on D 60; Table 3). The EDV (cm/sec) was unaffected by melatonin treatment (*P* > 0.05), but the scrotal circumference (cm) was slightly increased (*P*> 0.05) by the melatonin treatment at day 60 compared to day 0 since this elevation was non-significant.

**Table 3:** Testicular morphological echo-texture Doppler parameters, scrotal circumference and hormonal levels (estradiol17-ß + testosterone) in addition to nitric oxide expressed as mean ± SEM in Mel treated males compared to the normal ones at day 0 and day 60

Parameter	MEL treated (*n* = 6)		Normal control(*n* = 6)	
	D0	D60	D0	D60
Testicular volume	6.63 ± 0.02	7.02 ± 0.01	6.66 ± 0.01	6.98 ± 0.22
Scrotal circumference(cm)	11.01 ± 1.58	13.94 ± 1.25	13.25 ± 1.54	13.54 ± 1.65
PSV(cm/sec)	17.39 ± 1.84^a^	20.15 ± 0.99^b^	18.09 ± 2.14	18.32 ± 2.05
EDV(cm/sec)	4.04 ± 0.81	4.98 ± 0.01	3.66 ± 0.02	4.01 ± 0.04
Intra testicular colored area (pixels)	4985 ± 26.32^a^	5999 ± 45.21^b^	4865 ± 12.54	4556 ± 11.82
Testosterone (ng/mL)	6.22 ± 0.05^a^	8.98 ± 0.14^b^	7.02 ± 1.02	7.99 ± 0.04
Estradiol 17-ß (pg/mL)	32.15 ± 1.54^a^	55.21 ± 2.31^b^	26.54 ± 41.25	24.11 ± 11.32
NO (µmol/L)	37.11 ± 2.15^a^	64.25 ± 5.66 ^b^	62.24 ± 9.02	68.02 ± 10.21

Means with different (a, b) superscripts are significantly different at *P* < 0.05 within rows in MEL treated males

*PSV cm/sec* peak systolic velocity, *NO* Nitric oxide, and *EDV cm/sec* end diastolic velocity

The intratesticular colored area was increased in the MEL group at the end of examination (5999±45.21) compared to D0 of melatonin treatment (4985±26.32; Table 3), while in the normal males, the colored area value on D 0 was 4865±12.54 and on D 60 was 4556±11.82. In addition to testosterone, NO levels were elevated in MEL dogs at D 60 (8.98±0.14 for testosterone and 64.25±5.66 for NO). In contrast, estradiol 17-ß levels were elevated at D 60 (55.21±2.31) compared to D 0(32.15±1.54) in MEL dogs (Table 3). A negative correlation was noticed between distal artery Doppler indices and Doppler velocities, scrotal circumference, plasma testosterone levels, NO levels, and testicular volume in all dogs, as shown in Table 4.

**Table 4:** Correlation coefficients between testicular blood flow waveform (distal -supra- branch) measurements and semen analysis parameters in MEL dogs (*n* = 6)

Paired measurement	Pearson Correlation coefficients
DSTa PI × Scrotum circumference	-0.724^*^
DSTa PI × Testicular volume	-0.421^**^
DSTa PI × Intra testicular colored area	-0.405^*^
DSTa PI × Testosterone	-0.627^**^
DSTa PI × Estradiol 17-ß	0.711^**^
DSTa PI × NO	-0.499
DSTa PI × PSV	-0.742^**^
DSTa PI × EDV	-0.776^*^
DSTa RI × Scrotum circumference	-0.774
DSTa RI × Testicular volume	-0.848^*^
DSTa RI × Intra testicular colored area	-0.624^*^
DSTa RI × Testosterone	-0.455^**^
DSTa RI × Estradiol 17-ß	0.921^**^
DSTa RI × NO	-0.475^*^
DSTa RI × PSV	-0.844^*^
DSTa RI × EDV	-0.741^*^

^*^Means significant at 5%

^**^Means significant at 1%

*PI* pulsatility index, *RI* resistance index, *PSV* peak systolic velocity, *EDV* end diastolic velocity, *NO* nitric oxide, and *DSTa* Distal supra testicular artery

## Discussion

The pineal gland secretes an effective antioxidant hormone called melatonin, and this hormone acts as a direct scavenger of all free and hydroxyl toxic radicals and acts as an activator of some antioxidative enzymes such as glutathione peroxidase [44]. In addition, melatonin could protect DNA from damage under the effect of stress [45]. Furthermore, the melatonin hormone improved the process of spermatogenesis in animals when injected a few minutes prior to ischemia, and then after one day, melatonin improved the spermatogenesis process[46]. This finding aligns with our present study as we injected a single dose of melatonin and then assessed the morphometric measurements throughout 60 days. The semen characteristics, including (volume, concentration, total sperms×10^6^/ejaculation, and motility %) were increased in the MEL group, which concluded that melatonin injection could improve the semen quality and spermatogenesis process. Similarly, a study by Hemadi *et al.* [47] reported that one week of melatonin administration in the vitrified neonate testis that is grafted could play a critical role in the enhancement of the spermatogenesis through increasing the epithelium thickness [48].

Contradictory to our findings, another study revealed that melatonin did not affect semen quality and spermatogenesis [49]. However, this can be attributed to the different doses we used in the current study as well as the follow-up analysis every 15 days to demonstrate the effects on semen picture, as melatonin has a critical direct effect on male reproductive performance and share in testosterone synthesis from the Leydig cells in human [50] and animals [46].

In addition, melatonin-treated bulls exhibited an increase in semen picture and motility % by monitoring the hypothalamic-anterior pituitary testicular axis [51]. This enhancement may be due to the melatonin's primary effect on testicular blood flow, accompanied by increased steroid hormones ( estradiol and testosterone) and NO levels.

To our knowledge, the present study is the first to report the effect of subcutaneous injection of melatonin in dogs which a particular preference for the distal branch of supra testicular hemodynamics alterations. The relevant results proved the hypothesis that a single melatonin injection could improve testicular vascular perfusion through enhancement of the semen quality, steroidogenesis, and hormonal profile. This data is significant in improving male dog productivity and reproductive pattern [52].

The improvement of testicular blood flow by the melatonin injection led to a mark in both Doppler indices parameters of the distal supra testicular arteries (PI and RI), which have a strong negative correlation with blood velocities parameters (PSV and EDV), testicular volume and intra-testicular colored areas[53–55]. The marked linear decline in both two Doppler indices leads to a substantial decrease in the blood flow resistance pattern and, therefore, an elevation of the testicular blood supply with an increase in sufficient nutrients and oxygen within both testes [56–60].

The administration of melatonin in the form of an implant also leads to the stimulation of GnRH and testosterone levels [61, 62], as the melatonin action on the interstitial cells is related to the presence of melatonin-specific receptors in spermatogonial cells within the testis [63]. Melatonin is known to be involved in the production of estrogen from androgen via aromatase enzyme [64], and our present study reported a significant elevation of estradiol levels linked with a marked declination in both Doppler indices after melatonin administration. This finding can be attributed to the fact that estradiol has a vasodilatation action in testicular artery vascularization [65], in addition to the role of melatonin in the production of estrogen.

According to Zarlingo et al. [66], nitric oxide (NO), which is also evaluated by its blood metabolites (NOMs), plays a significant role in controlling blood flow [67, 68]. Due to the rapid inactivation of NO by reactive oxygen species (ROS), increasing NO levels in melatonin-treated dogs may increase the bioavailability of both NO and NOMs [69]. Melatonin may address this issue by preventing NO and ROS from reacting. Doppler ultrasonography in veterinary Andrology is still uncommon compared to human medicine. This restriction is attributable to two primary factors: the cost of portable devices and the second is the experts' lack of expertise. Finally, the angle of insonation that was impacted by the Doppler shift should be standardized in addition to the animal movement that may alter the Doppler reading measures

## Conclusion

This study revealed that one dose of melatonin increases testicular blood flow, triggers a significant rise in testosterone, estradiol, and nitric oxide levels, raises canine semen quality indicators, and decreases both Doppler readings of the distal supra-testicular artery.

## Supplementary Information

**Additional file 1.**

## Data Availability

All data collected or analyzed during this study are included in this published paper.
