# Supplementary material for: Single melatonin injection enhances the testicular artery hemodynamic, reproductive hormones, and semen parameters in German shepherd dogs
Source: BMC Vet Res. 2022 Nov 14;18:403. doi: 10.1186/s12917-022-03487-y (PMC9664593; doi:10.1186/s12917-022-03487-y)
Supplement: Supplementary file 1 — Additional file 1. [file 12917_2022_3487_MOESM1_ESM.pdf]

CERTIFICATE OF ENGLISH EDITING

BMC veterinary Research

THIS DOCUMENT CONFIRMS THAT THE MANUSCRIPT LISTED BELOW WAS EDITED BY AN ENGLISH LANGUAGE EDITOR WHO HOLDS A MASTER'S DEGREE IN APPLIED LINGUISTICS AND WORKS AS AN ASSISTANT LECTURER IN THE DEPARTMENT OF APPLIED LINGUISTICS AND INSTRUCTION (TEFL), AIN SHAMS UNIVERSITY, AND PH. D CANDIDATE AT UNIVERSITY OF ALBERTA, CANADA.

I HEREBY CONFIRM THAT ALL LINGUISTIC PROBLEMS HAVE BEEN ADDRESSED, AS WELL AS THE FOLLOWING ISSUES HAVE BEEN CORRECTED: GRAMMAR, PUNCTUATION, SPELLING, SYNTAX, PHRASING, STYLE AND SENTENCE STRUCTURE.

Manuscript Title

SINGLE MELATONIN INJECTION ENHANCES THE TESTICULAR ARTERY HEMODYNAMIC, REPRODUCTIVE HORMONES, AND SEMEN PARAMETERS IN GERMAN SHEPHERD DOGS

Authors

ALI SALAMA, ELSHYMAA A. ABDELNABY, IBRAHIM A. EMAM & MOHAMED FATHI

SIGNATURE

Marwa Abolfotouh

FOR CONTACT

abolfoto@ualberta.ca

Marwa.m.abolfotouh@women.asu.edu.eg

ENTER

DATE OF ISSUE

19-10-2022

PES

PROOFREADING AND EDITING SERVICES

MA119102022
